# Combining spatial and chemical information for clustering pharmacophores

**DOI:** 10.1186/1471-2105-15-S16-S5

**Published:** 2014-12-08

**Authors:** Lingxiao Zhou, Renate Griffith, Bruno Gaeta

**Affiliations:** 1 School of Computer Science and Engineering, UNSW Australia, Sydney, NSW, Australia; 2 School of Medical Sciences/Pharmacology, UNSW Australia, Sydney, NSW, Australia

**Keywords:** Pharmacophore comparison, Cluster analysis, Iterative closest point

## Abstract

**Background:**

A pharmacophore model consists of a group of chemical features arranged in three-dimensional space that can be used to represent the biological activities of the described molecules. Clustering of molecular interactions of ligands on the basis of their pharmacophore similarity provides an approach for investigating how diverse ligands can bind to a specific receptor site or different receptor sites with similar or dissimilar binding affinities. However, efficient clustering of pharmacophore models in three-dimensional space is currently a challenge.

**Results:**

We have developed a pharmacophore-assisted Iterative Closest Point (ICP) method that is able to group pharmacophores in a manner relevant to their biochemical properties, such as binding specificity etc. The implementation of the method takes pharmacophore files as input and produces distance matrices. The method integrates both alignment-dependent and alignment-independent concepts.

**Conclusions:**

We apply our three-dimensional pharmacophore clustering method to two sets of experimental data, including 31 globulin-binding steroids and 4 groups of selected antibody-antigen complexes. Results are translated from distance matrices to Newick format and visualised using dendrograms. For the steroid dataset, the resulting classification of ligands shows good correspondence with existing classifications. For the antigen-antibody datasets, the classification of antigens reflects both antigen type and binding antibody. Overall the method runs quickly and accurately for classifying the data based on their binding affinities or antigens.

## Background

Pharmacophore methods are widely used in drug discovery research projects [1]. As defined in the International Union of Pure and Applied Chemistry (IUPAC) glossary of terms [2], a pharmacophore describes chemical features and their spatial arrangement in active molecules and targets involved in specific biochemical interactions. Several software tools provide solutions for pharmacophore modelling and generation, including Accelrys Discovery Studio [3], LigandScout [4], ZINCPharmer [5].

Pairwise comparison of pharmacophores requires defining a similarity metric. Generally, there are two categories of similarity measurements: alignment-dependent methods and alignment-independent methods [6]. Alignment-independent methods usually target binary fingerprint descriptors, such as 3-point pharmacophore fingerprints [7] or 4-point pharmacophore fingerprints [8]. They calculate similarities with measurements such as the Tanimoto similarity (also called Jaccard Index as it was originally introduced by Paul Jaccard [9]). Alignment-dependent methods [6], in most of the cases, are based on shape or shape plus pharmacophore similarity measurements. Superimposition or overlays are widely used in this category of methods. However, chemical information is typically not involved in the shape-based methods. The OpenEye [10] colour-Tanimoto is an exception. It sums overlaps using customised pharmacophore features. However, this requires painstaking manual definition of the target features.

For grouping pharmacophores at a quantitative level, it is important to find an optimal partition method. Cluster analysis or clustering aims to separate data into groups or clusters. Clustering methods group data based on their pairwise distances. In other words, similar objects are grouped together more closely than dissimilar objects. There are some fundamental steps involved in a clustering activity including data extraction, similarity measurement, clustering and validation [11]. In cheminformatics applications, hierarchical clustering is one of the most popular approaches. These clustering methods group data based on their distances. The group average method (GA) and Ward's method [12] are two examples of hierarchical methods. Partition evaluation is a significant step to judge a clustering method. If the clustering method is applied to a benchmark dataset of known classification, then validation methods such as the Rand index and the adjusted Rand index [13] for supervised learning can be applied for comparing the results of the clustering method with the benchmark classification. Otherwise, unsupervised learning evaluation algorithms such as the Davies-Boulding index [14] can be used.

We present here a pharmacophore-aided Iterative Closest Point (ICP) clustering method for grouping pharmacophores using both their structural and chemical information. In this paper, Discovery Studio Modelling Environment http://accelrys.com, release 3.5 or 4.0, is used to generate the pharmacophores. There are six features defined in Discovery studio from which to construct pharmacophore models. They are Hydrogen bond acceptor, Hydrogen bond donor, Hydrophobic, Positive ionisable (from Catalyst's definition, a "Group that is, or can be, positively charged at physiological pH,") [3] , Negative ionisable (from Catalyst's definition, a "Group that is, or can be, negatively charged at physiological pH,") [3] and Ring aromatic (from Catalyst's definition, a "Five- or six-membered aromatic ring (vector)") [3]. A computer vision method, Iterative Closest Point (ICP) [15], is employed to calculate pharmacophore structural distances and a greedy alignment method is applied to measure the chemical distance. These two distance measures are then combined prior to hierarchical clustering. The method is evaluated relative to existing methods using two sets of experimental data. The results demonstrate that the proposed method is not only of benefit for classification of pharmacophores, but also has the potential to facilitate research in the field of antibody-antigen interactions.

## Methods

### Data preparation and pharmacophore generation

**Figure 1:**
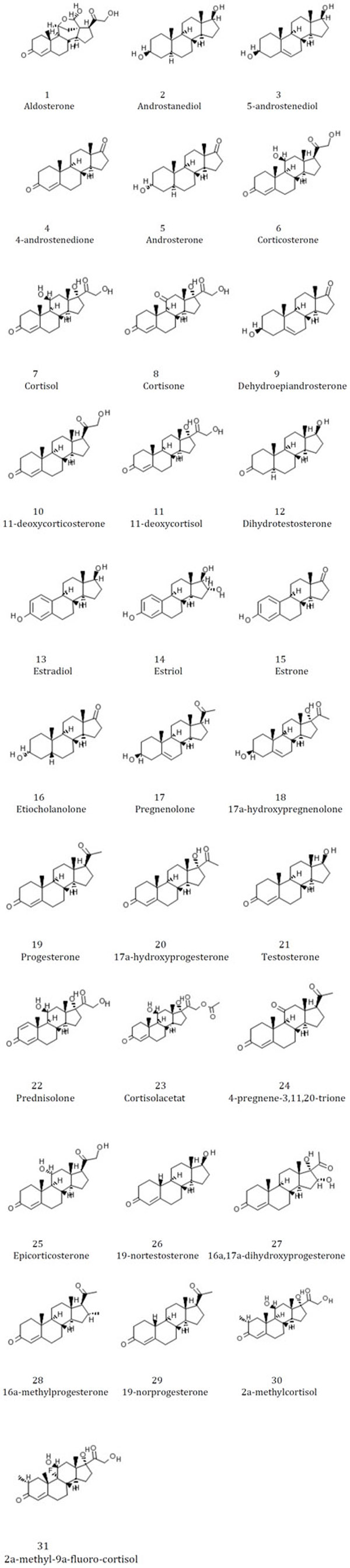
**2D molecular structures and names of the 31 globulin binding steroids**.

Two experimental data sets were used in testing. The first set of 31 globulin binding steroids (Figure 1) was introduced by Carmer *et al *[15]. In recent years, this dataset has been studied using a range of clustering methods and descriptors [16-19]. We compare our proposed method to a previous study [16] that used four-point pharmacophores as molecular descriptors.

Antibody-antigen binding is known to be highly specific [20]. Pharmacophores, by definition [2], can describe features involved in the interaction between compounds and target. Therefore, our second evaluation involves classifying pharmacophores generated from antibody-antigen complexes. The complexes were obtained from the Protein Data Bank [21] and information about the antibodies and antigens was gathered from an online self maintaining database SACS [22]. After applying the selection criteria (human sourced antibody-antigen complexes), 207 entries were selected and aligned by Clustal Omega [23]. To simplify the evaluation, 41 complexes were selected, corresponding to 3 differently named antibodies (17B, 2F5 and Anti-HIV V3 FAB 2557) and 2 types of antigens (GP120 and GP41) (See Additional file 1). However, Discovery Studio does not accept compounds over 1000 atoms or protein as ligands. Therefore, for each of the large (over 1000 atoms) protein antigens, the compound had to be cut into several parts and be saved in molecule format (SD file format). The cutting was based on the potential contact surface on the antigens. The potential contact surfaces were determined by finding the neighbouring (distance equal or less than 2.5 Å) amino acids of the antibody chains.

Discovery Studio Modelling Environment, release 4.0, generates the pharmacophores as *.chm files. Several protocols were employed for generating the pharmacophores. The autopharmacophore generation protocol selects pharmacophores using a Genetic Function Approximation (GFA) model [24]. This protocol aims to generate pharmacophore models from a single input molecule. Thirty one pharmacophores were generated using this protocol. The pharmacophore details for the globulin binding steroids have been recorded (See Additional file 2). For protein-ligand interactions, the GFA model as coded in the receptor-ligand pharmacophore generation protocol was used to produce structure-based pharmacophore models. Antibody-small molecules and antibody-protein parts were processed using this protocol. The details of the 41 antibody-antigen complex pharmacophores are listed in the table in Additional file 3. In this table, partial pharmacophores for large protein antigens were combined. The combination details are explained in the next section.

### Parsing pharmacophore files

The pharmacophore files produced by Discovery Studio include information such as name, coordinates, vector and tolerance etc. of the pharmacophore features. Based on our method, a set of Perl scripts were written to perform a series of steps to phase the pharmacophore files. Structural and chemical information was extracted from pharmacophore files. To simplify the calculation, some vector features, such as hydrogen bond donors and hydrogen bond acceptors were represented as one point. The coordinates of this point were provided by the centroid of the vector. Some statistics for each pharmacophore model were calculated and recorded, including the name of the features for each model, feature counters for each feature and so on. In the final stage of the phasing, the centroids of all pharmacophore models were normalized to (0,0,0), and new coordinates were calculated.

### ICP based structural distance calculation

The clustering was implemented in Matlab using the Iterative Closest Point (ICP) algorithm. ICP [15,25] is a method for optimizing the sum of squared distances between two sets of points. It is widely used in the fields of computer vision and robot navigation. The following is a summary of the ICP algorithm we implemented. It calculates the 3D structural information of two pharmacophores *p *and *q *to generate a rotation matrix R and a translation matrix T.

For k = 1 to k_max_

1. Do selection and matching Build k-d tree[26] and find closest neighbor pairs with KNN search

2. If matches to edge vertices or worst matches detected Do rejection point pairs

3. Weight matched points Weighting with compatibility of normal:

(1) $$\text{W}={\text{n}}_{p}*{\text{n}}_{q}$$

4. Minimize the error metric Calculate R with singular value decomposition (SVD)[27]:

(2) $$\text{R}=\text{V}*{\text{U}}^{\text{T}}$$

Calculate T:

(3) $$\text{T}=\bar{\bar{q}}-R*\bar{p}$$

5. Assign and apply transformation

End for

**Figure 2:**
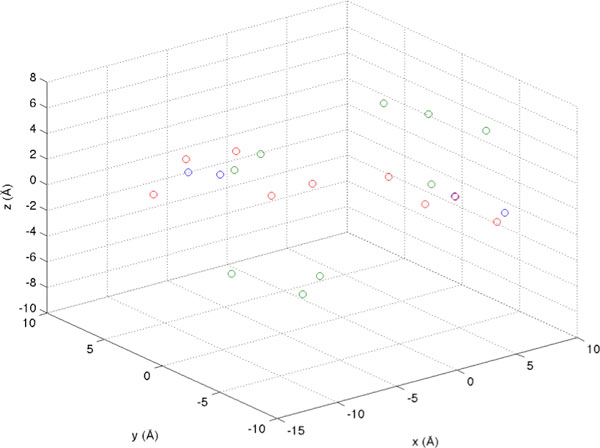
**ICP application to two antigens from PDB entries **1ADQ_P2[33]**and 3GBN **[34]. 1ADQ_P2 is shown in blue and is the reference model. Green points represent 3GBN before application of ICP. Red points correspond to 3GBN after ICP transformation based on 1ADQ_P2.

Figure 2. demonstrates this implementation by applying the ICP algorithm to our antibody-antigen dataset. Blue points represent the template set, the green and red points represent the second set, with the green points representing the initial pharmacophore locations and the red points representing them after application of the transformation.

The structural distance of the two pharmacophores was calculated using the Root-mean-square deviation (RMSD). RMSD values were normalized by dividing by the maximum distance. In the end, a N*N structural distance matrix was produced based on the number of pharmacophore models (N).

### Greedy alignment-based chemical distance calculation

The second significant part of the method is to compute a chemical distance matrix. A greedy alignment method was introduced to compare the chemical differences between pharmacophore models. This alignment approach was coded in Matlab like the ICP algorithm. In this method, a pharmacophore scoring matrix, as used in the Pharmacophore Alignment Search Tool (PhAST) [28], played an important role. The procedure of the greedy alignment is as follows. Let us consider two pharmacophore lists {*p*_i_} (pharmacophores 1) and {*q*_j_} (pharmacophores 2). *n *is the number of features in {*p*_i_} and *m *is the number of features in {*q*_j_}.

1. Find common features from both groups and remove them

2. Find the "best-unmatched" (feature pair with lowest dissimilarity score) features

a. Remove them

b. Increase the penalty score

3. Calculate gaps (|*n*-*m*|)

a. Increase the penalty score

The chemical distance matrix was calculated for each possible pair of pharmacophores. The matrix was then normalized by the maximum value of the gap penalty (by dividing each value in the matrix by the gap penalty * max(*n, m*)). A gap penalty score of 14 per position was used in the calculation, as in the PhAST method [28].

### Combined distance matrix

In the final step of the method, the structural distance matrix and the chemical distance matrix were integrated to form a mixed distance matrix. The combined matrix includes a geometric term S and a chemical term C:

(4) D=λ*S+(1-λ)*C

**Figure 3:**
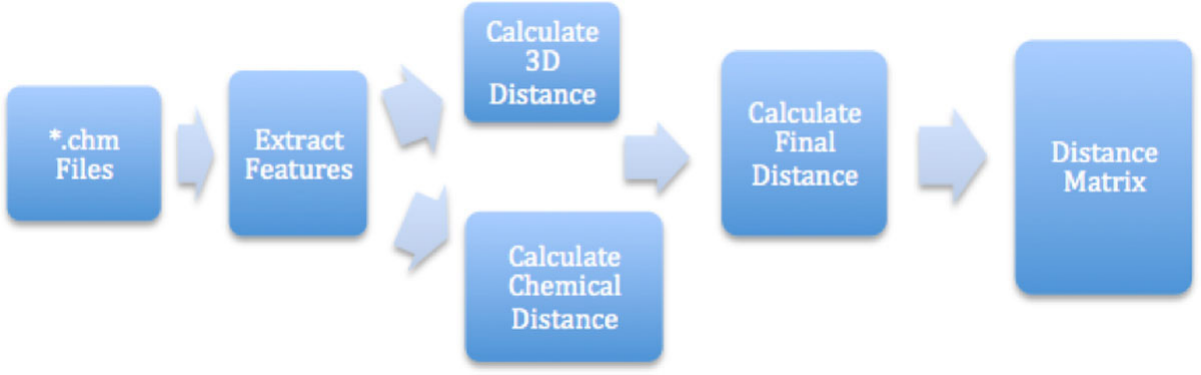
**Workflow of the ICP aided pharmacophore clustering method**.

In equation (4), λ can be adjusted to change the weights of 3D and chemical data. The workflow for the complete procedure can be found in Figure 3.

## Results

### Globulin-binding steroids

**Figure 4:**
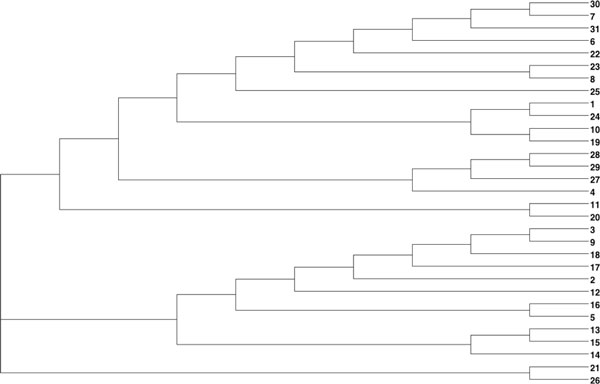
**Clustering of the 31 globulin binding steroids**. This dendrogram is showing the clustering of the 31 globulin binding steroids derived using a combination of 3D and chemical distances.

**Figure 5:**
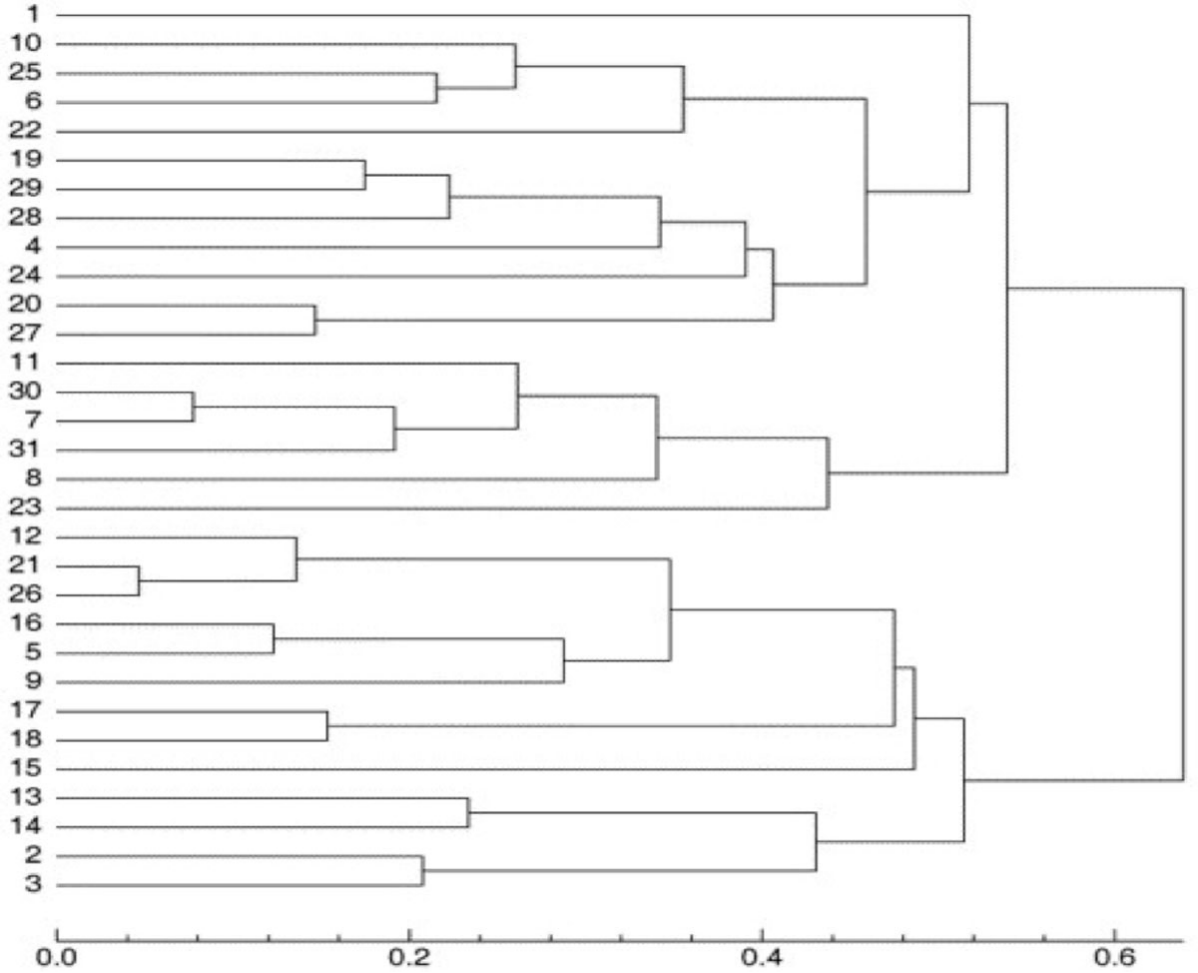
**Clustering of the 31 globulin binding steroids**. This dendrogram is showing the clustering of the 31 globulin binding steroids derived using the group average clustering method by Rodriguez [16].

**Figure 6:**
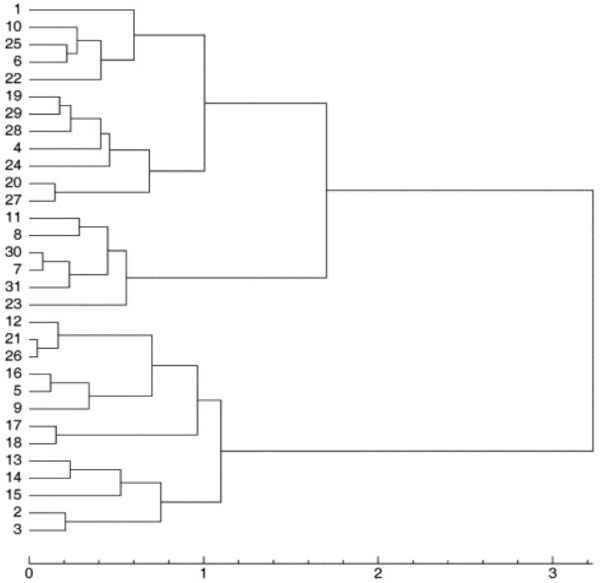
**Dendrogram showing the clustering of the 31 globulin binding steroids derived using Ward's clustering method by Rodriguez **[16]. This dendrogram is showing the clustering of the 31 globulin binding steroids derived using Ward's clustering method by Rodriguez [16].

After applying our clustering method, a 31*31 distance matrix was generated. The tree (Figure 4) was created using T-REX [29] from the combined matrix and using the neighbour joining method. This tree was compared with trees produced from the same dataset by two other methods [16]. One of the trees (Figure 5) was generated with the group average method [30], and the other one (Figure 6) was derived using Ward's method [12].

For further comparison, a table of binding affinity information for the 31 molecules from the literature [31] is provided as a gold standard to evaluate all three methods (Table 1). The 31 molecules were divided into two groups based on this binding affinity data: group 1 (CGB<-6.2) and group 2 (CGB>-6.2), to provide a reference clustering (Table 2) . For the clusterings produced by Rodriguez and that produced by our method, the 31 compounds were also labeled based on the clustering results (Table 2). Both clusterings were then compared to the reference CBG clustering using the Rand Index and adjusted Rand Index methods [13]. The evaluation results are shown in Table 3. All methods performed equally well in recreating the benchmark clustering.

**Table 1:** Binding affinities of the 31 globulin binding steroids [31].

Molecule	ID	CBG (pK_a_)
Deoxycortisol	11	-7.881

Corticosterone	6	-7.881

Cortisol	7	-7.881

Hydroxyprogesterone	20	-7.740

2a-methylcortisol	30	-7.688

Deoxycorticosterone	10	-7.653

Cortisolacetate	23	-7.553

Prednisolone	22	-7.512

Progesterone	19	-7.380

Epicorticosterone	25	-7.200

17a-methylprogesterone	28	-7.120

Cortisone	8	-6.892

19-Norprogesterone	29	-6.817

4-Pregnene-3,11,20-trione	24	-6.779

Testosterone	21	-6.724

Aldosterone	1	-6.279

16a,17a-Dihydroxyprogesterone	27	-6.247

19-Nortestosterone	26	-6.144

Dihydrotestosterone	12	-5.919

2a-methyl-9a-fluorocortisol	31	-5.797

4-Androstenedion	4	-5.763

Androsterone	5	-5.613

Pregnenolone	17	-5.225

Etiocholanolone	16	-5.225

Androstanediol	2	-5.000

5-Androstenediol	3	-5.000

Dehydroepiandrosterone	9	-5.000

Estradiol	13	-5.000

Estriol	14	-5.000

Estrone	15	-5.000

Hydroxypregenolone	18	-5.000

**Table 2:** Group labeling for 31 globulin binding steroids.

ID	CGB Grouping	R_GA	R_Ward	3D Clustering
1	1	1	1	1

2	2	2	2	2

3	2	2	2	2

4	2	1	1	1

5	2	2	2	2

6	1	1	1	1

7	1	1	1	1

8	1	1	1	1

9	2	2	2	2

10	1	1	1	1

11	1	1	1	1

12	2	2	2	2

13	2	2	2	2

14	2	2	2	2

15	2	2	2	2

16	2	2	2	2

17	2	2	2	2

18	2	2	2	2

19	1	1	1	1

20	1	1	1	1

21	1	2	2	3

22	1	1	1	1

23	1	1	1	1

24	1	1	1	1

25	1	1	1	1

26	2	2	2	3

27	1	1	1	1

28	1	1	1	1

29	1	1	1	1

30	1	1	1	1

31	2	1	1	1

The labels for the 31 globulin binding steroids are based on CBG (binding affinity), Group Average (R_GA), Ward (R_Ward) (Rodriguez [16]) and the combined 3D distance and chemical distance method proposed in this study (3D Clustering).

**Table 3:** Evaluation of different clustering methods for 31 globulin binding steroids.

Method	Rand Index (0 to 1)	Adjusted RI (-1 to 1)
R_GA	0.8194	0.6387

R_Ward	0.8194	0.6387

3D Clustering	0.8194	0.6378

Rand index and adjusted Rand index are given for the comparison of each of the clustering methods with the reference CBG clustering for 31 globulin binding steroids.

### Antibody-antigen complex dataset

**Figure 7:**
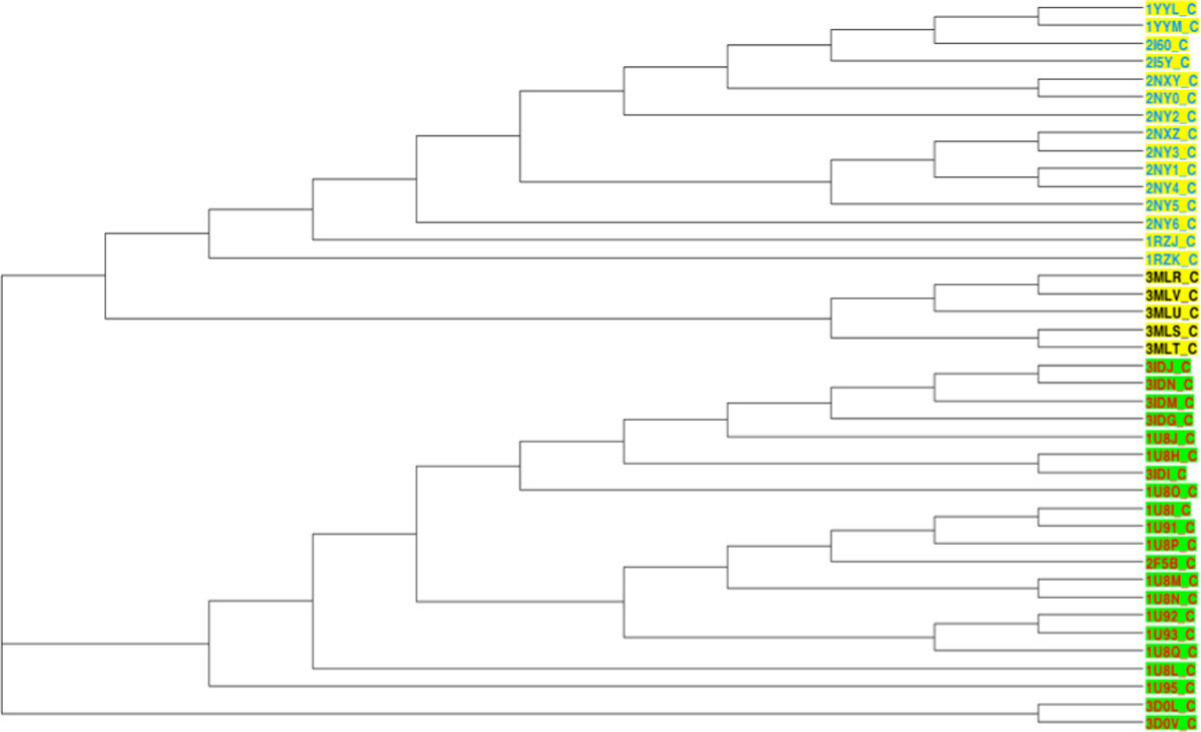
**Clustering of 41 antibody-antigen complexes based on combined distance**. This dendrogram is showing the clustering of four groups of antibody-antigen complexes based on a combination of 3D and chemical distances. Complexes with antigen GP41 are shown with a green background. Complexes with antigen GP120 are shown with a yellow background. Complexes with antibody 17B, are shown with their PDB ID colored blue. Complexes with antibody 2F5 are shown with their PDB ID colored red. Complexes with antibody ANTI-HIV-1 V3 FAB 2557 are shown with their PDB ID colored black.

In this section, the ICP-based pharmacophore-aided method was applied to classify 4 groups of pharmacophores. The clustering method generated a 41*41 distance matrix. T-REX translated the distance matrix into a dendrogram (Figure 7).

To evaluate the result, we categorised the 41 complexes into two groups based on their antigens, as a benchmark clustering. Results from the new method were clustered into 4 groups (Figure 7 and Additional file 1). There were two large clusters G1 (antigen GP41), G2 (antigen GP120). Complexes 3D0L and 3D0V were misclassified, so we labelled them as G3 (3D0L) and G4 (3D0V). These two classifications were compared using the Rand Index and Adjusted Rand Index. The results (Table 4) demonstrate an excellent agreement between the two classifications.

**Table 4:** Evaluation of 3D plus chemical clustering method for antibody-antigen complexes.

Method	Rand Index (0 to 1)	Adjusted RI (-1 to 1)
3D Clustering	0.9524	0.9046

Rand index and adjusted Rand index results for comparison of the 3D plus chemical clustering method with the reference clustering based on antigen identity for 41 antibody-antigen complexes.

## Discussion

In the dataset of 31 steroid compounds, some pairs had been reported that should be grouped together closely ([16]). They were (21, 26), (7, 30) and (19, 29), that differ only by a methyl group. Molecules 5 and 16 only differ by the stereochemistry of one centre on the A ring. Comparison of Figures 4, 5 and 6 demonstrate that all three different methods have successfully grouped those reported pairs. The special structures of the two compounds 4 and 31 led to a misclassification (they were classified into group with pKa < -6.2) in all three methods. Molecules 21 and 26 were incorrectly clustered as an exceptional cluster by our new method. With the exception of those molecules (21 and 26), the group average method, Ward's method and our method all produced trees with the same two superclasses. Rodriguez's methods and the new method have the same Rand Index value and a very close adjusted Rand Index. Additionally, all Rand Index and adjusted Rand Index scores are above the threshold for a 'good' clustering (0.5 for Rand Index, 0 for adjusted Rand Index).

**Figure 8:**
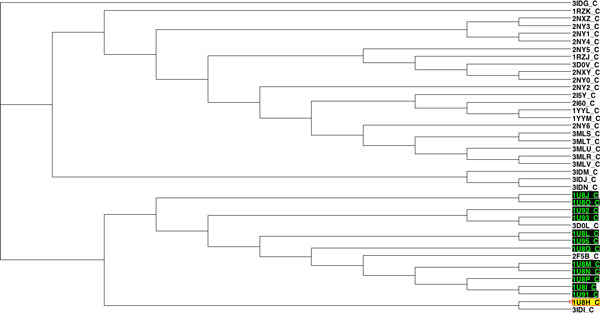
**Clustering of 41 antibody-antigen complexes based solely on 3D distance**. This dendrogram is derived from a classification of antigens in a set of 41 antibody-antigen complexes based solely on 3D distance. 1U8H (yellow highlight) is structurally different from other similar complexes and is clustered separately from the other 1U8* complexes (black highlights).

**Figure 9:**
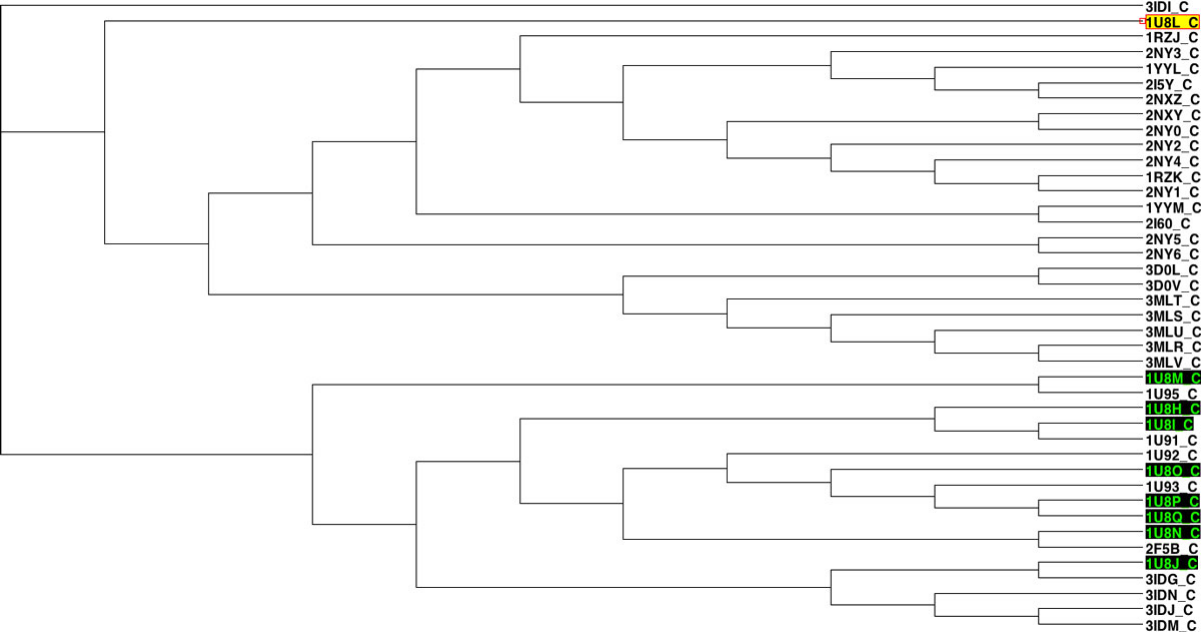
**Clustering of 41 antibody-antigen complexes based solely on chemical distance**. This is a dendrogram based on a classification of antigens in a set of 41 antibody-antigen complexes based solely on chemical differences between pharmacophores. 1U8L (yellow highlight) chemically differs from other similar complexes and is clustered away from the other 1U8* complexes (black highlights).

**Figure 10:**
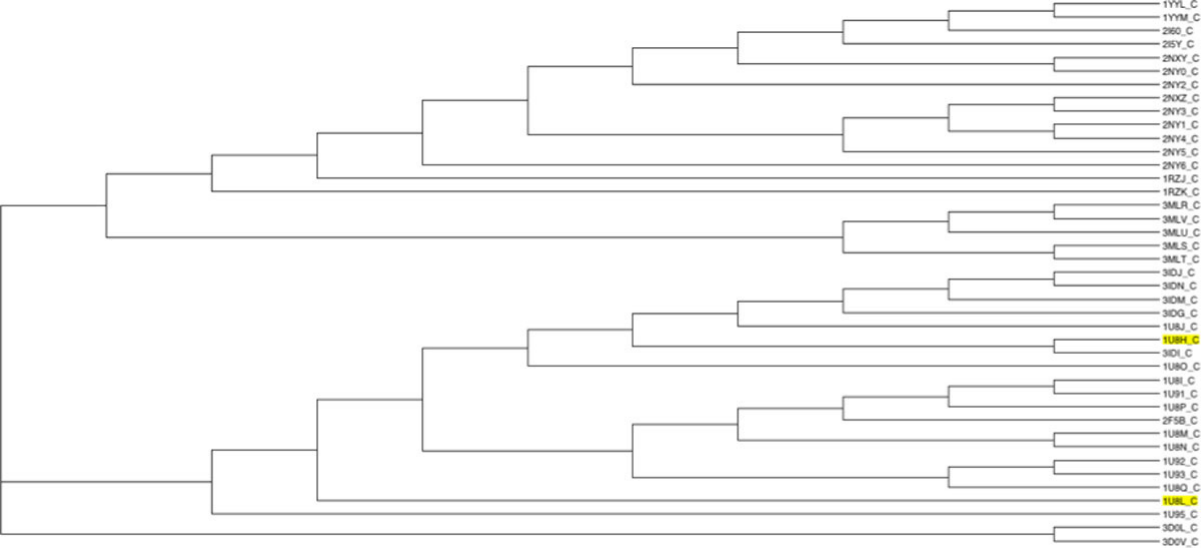
**Clustering of 41 antibody-antigen complexes based on combined distance**. This section of a dendrogram calculated from a distance measure combining 3D structural distance and chemical distance between pharmacophores. 1U8L and 1U8H (highlighted in yellow) are correctly identified based on antigen.

Considering the application of the proposed method to 41 antibody-antigen complexes, the pharmacophores were generally classified into two large super-clusters based on their antigens. One supercluster included all complexes with GP41 or a GP41 analog as antigen. The second supercluster had all the complexes with GP120 or one of its fragments as antigen. The classification did not only identify the antigens, within each supercluster, pharmacophores also formed clusters corresponding to their binding antibody (e.g. G1 with 17B as antibody and G4 with ANTI-HIV-1 V3 FAB 2557). Additionally, the Rand Index and adjusted Rand Index both were very high, which means the ICP aided method performed well in clustering. In addition some interesting structural and chemical features highlighted by other researchers could be identified in the results. In complex 1U8H, the Glu662 substitution has been reported to involve a water network rearrangement and thus this complex is structurally different from the other 1U8* complexes [32]. This can be seen by the unexpected position of 1U8H in a clustering based solely on 3D distance calculated using ICP (Figure 8). In the same paper, 1U8L was reported to have chemical differences to the other 1U8* complexes. This can be seen on a dendrogram based solely on chemical distances (Figure 9). However, when 3D and chemical distances were combined, 1U8L and 1U8H were correctly clustered with other complexes with similar antigens (Figure 10).

## Conclusions

A method combining a structural distance based on ICP and a "chemical" distance has been developed and has been demonstrated to successfully partition pharmacophores based on the types of antigens in a set of antibody/antigen complexes or binding affinity in a set of steroids. In addition, the method is very fast. The 41 pharmacophore comparison only took around 30 seconds on a desktop computer (Apple iMac, 2.7 GHz Intel Core i5, 8GB Memory). However, the method requires the number of pharmacophores being compared to be similar and was less accurate when the following ratio was larger than 2.: Max(Number_of_Pharmacophores)/Min(Number_of_Pharmacophores)

## Competing interests

The authors declare that they have no competing interests.

## Authors' contributions

All authors read and approved the final manuscript.

## Supplementary Material

Additional file 1 **Antibody-antigen complexes**. This table summarises antibody-antigen complexes used in this study with their cluster number as assigned by the ICP-based method. Click here for file

Additional file 2 Number of pharmacophore features in the 31 globulin binding steroids used in this study Click here for file

Additional file 3 Number of pharmacophore features in the 41 antibody-antigen complexes used in this study Click here for file
